# E-Learning in Nursing and Midwifery during the COVID-19 Pandemic

**DOI:** 10.3390/healthcare11233094

**Published:** 2023-12-04

**Authors:** Nataša Mlinar Reljić, Maja Drešček Dolinar, Gregor Štiglic, Sergej Kmetec, Zvonka Fekonja, Barbara Donik

**Affiliations:** 1 Faculty of Health Sciences, University of Maribor, Žitna ulica 15, 2000 Maribor, Slovenia; gregor.stiglic@um.si (G.Š.); sergej.kmetec1@um.si (S.K.); zvonka.fekonja@um.si (Z.F.); barbara.donik@um.si (B.D.); 2 Health Center Celje, Gregorčičeva ulica 5, 3000 Celje, Slovenia; maja.cdolinar@gmail.com

**Keywords:** e-learning, nursing care, midwifery, pandemic

## Abstract

As the COVID-19 pandemic continues to spread, e-learning has increased. This is a challenge for nursing and midwifery students, as clinical training is an essential part of their education. The aim of this review was to identify the advantages and limitations of e-learning for nursing and midwifery students during the COVID-19 pandemic. A systematic review of the literature was conducted following the PRISMA guidelines. The international databases PubMed, CINAHL/MEDLINE, Web of Science, and ScienceDirect were searched. Articles were critically appraised. Thematic analysis was used to synthesise the data. The search resulted in 91 hits. Thirteen studies were included in the final analysis. Three main themes were identified: (1) the benefits of e-learning; (2) the challenges/limitations of e-learning; and (3) recommendations for e-learning. E-learning in nursing and midwifery is an effective alternative learning process during the COVID-19 pandemic. Students perceive several benefits and challenges related to internet access, technical equipment, financial aspects, and work and family commitments.

## 1. Introduction

Before the COVID-19 pandemic, e-learning was becoming an increasingly common form of education, albeit less common in health sciences such as medicine, dentistry, midwifery, and nursing. E-learning refers to a course when the learning has been partially or fully taken online, using information and communication technology, eBooks, text messages, and videos, links, and discussions [1].

During the COVID-19 pandemic, countries worldwide began to use e-learning for students. Faculties were trying to make the most of the technological transformation and provide a better learning environment for students. E-learning has witnessed a massive boom, as it allows flexibility in studying and options to determine a convenient time frame and study space [2]. It also allows distance learning materials, ease of management, and accessibility [3,4].

E-learning students face different challenges during the COVID-19 pandemic [5]. The sudden leap from traditional education to complete e-learning, careless planning and inadequate training on e-learning are the challenges of e-learning during COVID-19 [6]. Many faculties enabled students to use online resources and published lectures online. In contrast, some faculties were not sufficiently prepared for the unexpected transition and implementation of the curriculum in an e-environment [7]. The lack of information communication technology (ICT) resources [8] and difficulties in maintaining academic integrity [3] also provided additional challenges for effective e-teaching. In addition, there are doubts about whether e-learning in nursing and midwifery can improve learning outcomes compared to traditional approaches in education [9]. Iyer et al. [10] concluded that no virtual lecture could replace the experience of working with patients. E-learning offers flexible learning methods, although nursing and midwifery students may have a negative attitude towards e-learning compared to face-to-face lectures [11,12]. McDonald et al. [13] stated that e-learning alone cannot replace face-to-face learning. Other weaknesses of e-learning, as stated by Olivera et al. [14], can be self-discipline and organisation, which are a problem for students who cannot fulfil the required study obligations without the physical presence and supervision of the teacher. In addition to the technical difficulties and overload of the online environment due to e-learning, students also report fatigue, anxiety and worry due to the overuse of virtual communication platforms. Researchers dubbed this “zoom fatigue” [15]. According to Suleyiman and Zewdu [16], overloads, such as inappropriate task timing, learning difficulties, procrastination, and time management problems, can lead to high levels of stress or strain.

This systematic review aimed to identify the advantages and limitations of e-learning for nursing and midwifery students during the COVID-19 pandemic. The research question was: How does existing evidence explain the benefits and limitations of e-learning for nursing and midwifery students during the COVID-19 pandemic?

## 2. Materials and Methods

A systematic review was conducted using a literature review method, analysis and synthesis, and the method of compilation [17]. The review included original quantitative, qualitative and mixed-methods studies. The Preferred Reporting Items for Systematic Reviews and Meta-Analyses (PRISMA) checklist was used [18] for presenting the results.

### 2.1. Search and Selection Strategy

The literature search was conducted in four international databases (PubMed, CINAHL, ScienceDirect and Web of Sciences) in the spring of 2022 using predefined search terms according to the PI(C)OT (Participants, Intervention, Outcome, Time) inclusive and exclusion criteria [19]. The search terms included: nursing student, midwifery student, Coronavirus COVID-19, COVID-19 pandemic, COVID-19 epidemic, SARS-CoV-2, e-learning, distance learning, online learning, e-education, e-training, online education, experience, benefit, advantage, impact, problems, and effectiveness. The search terms were combined using synonyms and Boolean operators. Studies were eligible if they met the following criteria: (1) population: nursing students, midwifery students; (2) intervention: e-learning; (3) outcome: benefits, limitations, effectiveness, success, experience with e-learning; and (4) study design: primary quantitative, qualitative or mixed-methods studies evaluating e-learning during the COVID-19 pandemic. The following exclusion criteria were used: articles published in languages other than English, anecdotal reports, opinions, systematic reviews, and editorials about e-learning during the COVID-19 pandemic. The published sources were time-limited from December 2019 to spring 2022. Three authors independently conducted the search process, two more authors performed the quality assessment, and then the researchers discussed the results till agreement. The researchers agreed on the 13 included studies in the thematic analysis.

### 2.2. Quality Appraisal

The Joanna Briggs Institute (JBI) tools were used to evaluate the studies. The JBI Qualitative Assessment Research Instrument was used for the qualitative studies, and the Critical Appraisal Checklist for Analytical Cross-Sectional Studies or the Critical Appraisal Checklist Cohort Studies were used for studies based on quantitative methods. To assess the article quality in qualitative studies, we considered a scoring system. The three categories were: “low quality” (0–4), “medium quality” (5–7), and “high quality” (8–10). For cross-sectional studies, the scores determined “low quality” (0–2), “medium quality” (3–5), and “high quality” (6–8). Cohort studies yielded “low quality” (0–4), “medium quality” (5–8), and “high quality” (9–11) categories [20].

The Mixed Methods Appraisal Tool (MMAT) was used for the quality assessment of the mixed-methods studies [21]. The MMAT classification predicts five quality scores: very low (0%), low (25%), moderate (50%), high (75%), and very high (100%) [21].

### 2.3. Data Extraction and Analysis

Data were extracted from all the included studies: author, year of publication, country, purpose, participants, research design, and main findings. The thematic analysis was performed according to Thomas and Harden’s [22] guidelines. In the first step, the text was read line by line and the free codes in the article identified. The identified free codes were then organised into a subtheme and named according to the thematic characteristics. The subthemes were grouped into main themes and named [22]. Three authors carried out the thematic analysis and synthesis. Disagreements were resolved through discussion.

## 3. Results

**Figure 1:**
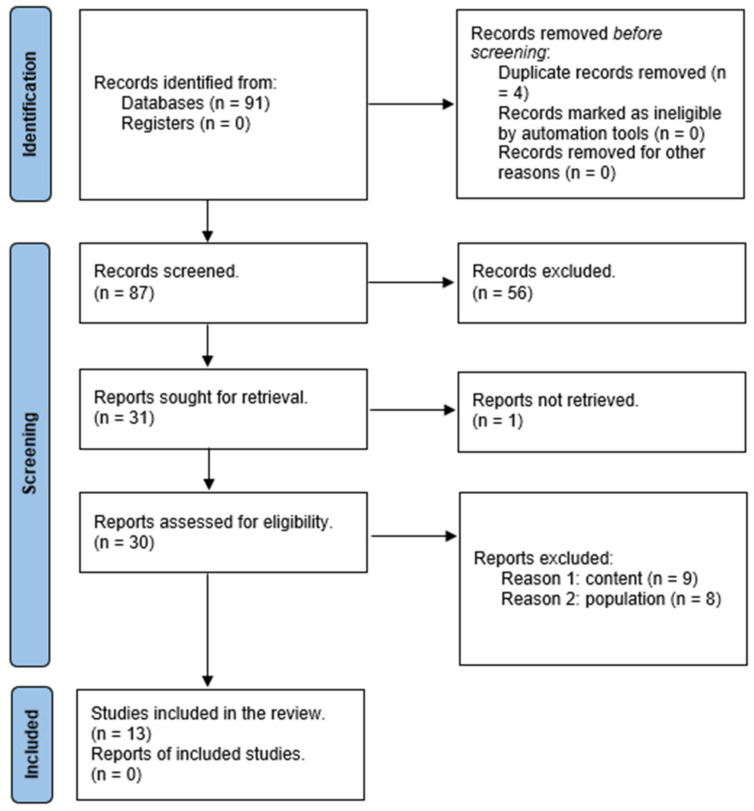
PRISMA flow chart of the selection process.

This section presents the results of the search process, the characteristics of the study and the thematic analysis. The search process yielded 91 sources. Seven records were retrieved from the PubMed database, one from CINAHL, seventeen from Web of Science and sixty-six from ScienceDirect. Duplicates (*n* = 4) were found and eliminated using Mendeley Reference Management Software. Then, the records (*n* = 87) were screened and excluded as not appropriate according to the predefined inclusion criteria. Articles (*n* = 30) were selected for eligibility by reading the full text. In total, 13 studies were included in the final analysis (Figure 1).

### 3.1. Study Characteristics

The main characteristics of the articles are presented in Table 1. Of the thirteen included articles, only three surveys were conducted in Europe, two in Spain [23,24] and one in Sweden [25]. Three surveys were from Turkey [26,27,28]. Two surveys were conducted in the Philippines [29,30], and the other surveys were conducted in the USA [31], Egypt [12], Zambia [32], Jordania [33], and Ghana [34]. The total sample of research participants represents 2957 students. The methodological characteristics show that seven surveys were based on quantitative methodological design [12,23,26,27,29,30,34], two surveys used mixed-methods [25,35] and four surveys were conducted using qualitative design [24,28,31,32].

### 3.2. Study Quality Appraisal

The study quality appraisal results show that three studies [23,30,32] were medium quality (Table 2, Table 3 and Table 4). Eight studies [12,24,26,27,28,29,31,34] were scored as high quality (Table 2 and Table 3). Studies using mixed methods were assessed as high [25] and very high quality [33], as presented in Table 5.

### 3.3. Advantages and Limitations of E-Learning in Nursing and Midwifery during the Pandemic

Three main themes emerged from the data analysis and synthesis: (1) advantages of e-learning; (2) challenges/limitations of e-learning; and (3) recommendations for e-learning, as presented in Table 6.

#### 3.3.1. Advantages of E-Learning

The findings of this study emphasise the crucial role of e-learning as a consequential and efficacious alternative in the field of nursing and midwifery education during the COVID-19 pandemic. E-learning emerges as a facilitator, allowing students to seamlessly integrate academic pursuits [25,32], familial responsibilities, and professional commitments [24]. It will enable students engaging with recorded lectures repeatedly, thereby accommodating individuals requiring extended study durations and contributing to favourable academic outcomes [28,31]. Notably, e-learning instigates a heightened sense of responsibility among nursing and midwifery students, with a discernible enhancement of their time-management and information-retrieval skills [25,31].

Moreover, the financial economy of e-learning is evident, manifesting in the conservation of resources otherwise expended on commuting. Simultaneously, the time savings from eliminating travel are redirected towards familial responsibilities [25,31]. The study reveals a positive correlation between students’ technological dependence, underscored by the prevalence of smartphones among the student cohort [34], and their favourable disposition towards e-learning [26,29].

Furthermore, e-learning presents an ancillary benefit in mitigating health risks, specifically the reduction of exposure to COVID-19, by enabling students to engage in learning within the safety of their homes [28]. This dual advantage of enhanced safety and technological integration contributes significantly to the viability and desirability of e-learning platforms in the context of nursing and midwifery education during the pandemic.

#### 3.3.2. Limitations/Challenges of E-Learning

Long-time exposure to e-learning burdened nursing and midwifery students and caused high anxiety levels [23], stress [30] and sleep disturbances [31]. It was also found that students feel isolated, lonely and miss their peers [31]. The sudden transition to e-learning without prior preparation was particularly burdensome and reduced socialisation opportunities [25].

Implementing e-learning engendered heightened daily academic demands for students, notably those enrolled part-time, for whom the virtual learning environment induced significant stress. The confluence of employment-related commitments constrained their ability to engage fully in e-study. Furthermore, the strain was exacerbated by familial obligations, as changes in family dynamics introduced additional burdens, particularly for part-time students in contexts where caregiving responsibilities traditionally fall within the purview of women [24].

An in-depth review of the data collected reveals several challenges related to students’ infrastructure. Predominantly, disruptions and/or suboptimal internet connectivity emerged as pervasive impediments, a recurring theme highlighted across multiple data sources [12,24,25,26,31,32,34]. These challenges highlighted the multifaceted obstacles students face navigating the e-learning landscape.

E-learning is also influenced by the space available to students at home. The lack of privacy and adequate space for studying presented challenges, especially for students in environments where families are large [33] or the conditions of their home environment do not allow high-quality e-learning [24,31].

Good relationships and communication are essential for a successful learning process between lecturer and student. Body language and eye contact [31] contribute to a better understanding of the content and study motivation [25,29]. The results show that e-learning does not allow quality interaction between lecturer and student like face-to-face teaching does. Reflection and feedback with explanations are often missing [24]. In the e-learning process, students are reluctant to ask questions during lectures because they do not know if the lecturer can hear them, are ashamed to ask, or do not know if the connection is working well [31].

#### 3.3.3. Recommendations for E-Learning

The findings show that faculty anxiety about using online tools and student anxiety about technology during exams can be significantly decreased by targeted training interventions, thereby improving the efficacy of e-learning [31]. Nursing and midwifery e-learning shows increased effectiveness and efficiency when clinical training is conducted in healthcare settings [24,33]. The majority of clinical training must be conducted in authentic clinical environments, as reliance on video-based training methods does not ensure the attainment of practical skills [33].

The findings emphasise that recorded lectures emerge as the most effective modality, facilitating iterative review [28,31]. Videoconferencing, pre-recorded lectures, assignments, materials in PPT and PDF formats [24], and instructional videos encapsulating learning content [34] are advocated for in terms of the effective implementation of e-learning for nursing and midwifery students. The fundamental prerequisites for successful e-learning include robust internet connectivity and the use of suitable technology to ensure uninterrupted educational experiences [34]. Equitable access to information and communication technology is crucial for students and lecturers [24].

Students must take care of their psychophysical, social, and spiritual well-being during e-learning. Spending time in the garden [23] and physical activity [31] can help students maintain their well-being.

## 4. Discussion

This systematic review presents the advantages and limitations of e-learning for nursing and midwifery students during the COVID-19 pandemic. Our findings show that e-learning offers some benefits. Nursing and midwifery students could adjust to listening to lectures, listen to lectures repeatedly, save time because of no travelling needs, and take more responsibility for their studies. In addition to the many advantages, midwifery and nursing students faced challenges like social isolation brought about by e-learning during the pandemic. Unique challenges associated with clinical training in the context of e-learning underscored the need for innovative solutions in terms of practical skill acquisition. The evolving landscape of teaching methodologies in the digital realm necessitated reevaluating and adapting traditional instructional approaches to align with the nuances of e-learning. Both academically and personally, strategies for coping with the increased burdens imposed by e-learning emerged as a crucial area for exploration, suggesting avenues for support and intervention. E-learning during the COVID-19 pandemic increased loneliness and social isolation among nursing and midwifery students. Both pose a significant threat to mental health and well-being.

In 2020, the world faced the COVID-19 pandemic, which affected healthcare, social institutions, and society [35]. Also, the higher education institutions that educate nursing and midwifery students have faced unique challenges. Many healthcare institutions withdrew students from the clinical environment [36]. This represented a significant educational challenge, as clinical training represents half of the entire educational process in an undergraduate nursing study programme. Most of the study obligations have been moved to an online learning environment. E-learning represents an alternative approach to future nurses’ and midwives’ education. Such an approach moves the student from a passive listener to an active participant and promotes innovative and critical thinking [37]. While some researchers [38] note that e-learning provides interactivity and encourages collaboration and exchange of ideas between students and teachers, our results did not confirm this. Our findings show that face-to-face contact, body language expressions and eye contact enable building a relationship between student and lecturer and significantly impact study motivation. Face-to-face attendance and teacher–discussant contact were also important in some research [39].

E-learning saves students and teachers time they would have spent travelling [25] and enables the allocation of study time according to their preferences and adjusting to other obligations, such as family and work [24,31]. In addition, we note that e-learning is comfortable for students, allows easy access to learning materials at any time and makes it easy to use, which is confirmed by other authors [2,3]. Furthermore, positive experiences with e-learning can contribute to learning satisfaction and engagement [40].

Despite its many advantages, e-learning presents some challenges and limitations [2,24,35]. Among these, social isolation and loneliness should be mentioned, as reported in some other research [2,41,42,43]. Nursing and midwifery students have had considerable challenges with increased workloads [24,44,45], which resonate with the findings in this review. This is particularly pronounced for students who are employed. The workload made studying difficult. In addition, family loads joined workloads when students cared for their children. Most nursing and midwifery students are female and perform most of the care for the family. We also highlight economic factors that have affected e-study during the COVID-19 pandemic as essential challenges. For students with a weaker social status, e-learning was a limitation. We see the reason for this in the lack of technical equipment and access to the internet, which other research also warns of [35]. Many health problems arose among students due to e-learning during the COVID-19 pandemic, like problems with concentration, problems with sleep, vision, and pain, such as headaches, neck pain, back pain, anxiety and stress [44,45,46,47]. We found social isolation can be a problem for some students. Still, on the other hand, e-learning is suitable, especially for students who do not want many social interactions or who find it challenging to establish interpersonal relationships [46].

In the following, we offer some recommendations for effective and successful e-learning in nursing and midwifery. Adequate technical and infrastructural equipment with access to the online network is imperative for e-learning. Clinical training represents the core of the nursing and midwifery profession; therefore, it must be provided in the clinical environment, not only in simulated environments. Clinical training imbues students with confidence and professional competence for nursing and midwifery practice. Nevertheless, e-learning could be considered an alternative nursing and midwifery education approach. It could be used for teaching learning modules or complementing face-to-face training in training programmes [48]. We also recommend taking care of students’ mental health to prevent anxiety and depression because of an absence of interpersonal communication. University staff can contribute to avoiding and increasing students’ mental health issues by maintaining a stable educational framework, providing high-quality distance teaching and encouraging and supporting students through the pandemic period [49]. We believe stress management programmes are necessary for nursing and midwifery students to help foster mental health coping skills.

It would be also interesting for future research to evaluate e-learning’s effectiveness compared to traditional learning in-person.

This literature review has some limitations. In the search strategy, we limited the research articles to English and excluded the remaining articles in other languages, which could affect the analysis. Studies, mostly conducted outside Europe, were included in the review, representing a limitation of the implementation and use of the findings in the education of nurses and midwifery in Europe.

## 5. Conclusions

The COVID-19 pandemic has caused a sudden shift from traditional classroom teaching to e-learning. E-learning brings new challenges for both students and teachers.

It has many advantages, such as safety against SARS-CoV-2 infection, greater accessibility to educational content independent of time, independence and greater student innovation in studies, saving time and financial resources, adjustment of study time and more time for family.

However, some limitations and challenges were also revealed. The limitations present increased burdens in terms of coordinating private life and studies, inadequate or deficient information and infrastructure support. Furthermore, poorer social contact with fellow students, health problems related to e-learning, the financial burden that comes with the purchase of new telecommunications equipment, inadequate space and lack of privacy and communication problems present challenges to e-learning.

Experience with e-learning brought new knowledge, insights and feelings that can encourage further research and improvements in this area.

## Figures and Tables

**Table 1:** Main characteristics of the studies.

Reference (Author/Year)	The Objective of the Study	Research Design	Sample	Main Findings	
				Advantages of E-Learning	Limitations of E-Learning
Alhassan (2020), Ghana [34]	To determine the preparedness and feasibility conditions among nursing and midwifery students regarding patterns of use of e-learning applications and social networks for learning purposes.	Quantitative methodology; cross-sectional descriptive design.	*n* = 233 nursing and midwifery students.	All students owned smartphones, mainly used for internet voice, phone calls, social network texting and short messaging services. In addition, 60% of students used smartphones for accessing e–learning content.	Students experience a lack of seamless internet connectivity, low bandwidth availability and poor telecommunication and ICT infrastructure quality within their learning environments to maximise the benefits of e-learning opportunities.
El-Hamed Diab and Elgahsh (2020), Egypt [12]	To investigate the effect of obstacles faced by nursing students on their attitudes towards e-learning while applying it during the COVID-19 pandemic.	Quantitative methodology; descriptive correlational design.	*n* = 627 students (192 first-year, 160 the second year, 144 in the third year and 131 fourth-year students).		Some 62% of students had negative attitudes towards e-learning. The highest level of obstacles that nursing students faced during e-learning presented among students in the first year (68%), and the lowest level of total obstacles present among students in the fourth year (56%) The most common dimensions of obstacles in e-learning were infrastructure and technology, technical and management support, and instructors’ characteristics. There was a high statistically significant negative correlation between obstacles facing nursing students and their attitudes toward e-learning.
García-González et al. (2021), Spain [23]	To explore the influence of the COVID-19 pandemic on the anxiety levels of nursing students because of e-learning.	Quantitative methodology; cohort cross-sectional design.	*n* = 460 nursing students.	Students living in a house with a garden reported a lower level of anxiety than students residing in apartments without a garden.	Significant predictors of anxiety in students are the final year of study, female gender, and living in an apartment/house without a garden. Most students were emotionally affected by anxiety. The level of anxiety in the fourth week increased compared to the first week.
Guven Ozdemir and Sonmez (2020), Turkey [26]	To determine the relationship between nursing students’ levels of technology addiction and their attitudes toward e-learning during COVID-19.	Quantitative methodology; cross-sectional design.	*n* = 434 nursing students.	A positive correlation was found between students’ addiction to technology and their attitudes towards e-learning. Nursing students only had a partially positive attitude towards e-learning due to the limitations of acquiring practical skills.	A limitation of e-learning was the availability of an internet connection. Some 65% of students believe that e-learning is not effective.
Langegård et al. (2021), Sweden [25]	To describe and evaluate nursing students’ experiences of the pedagogical transition from traditional campus-based learning to distance learning using digital tools.	Mixed-methods study; exploratory sequential design.	*n* = 96 nursing students.	Most students prefer the traditional learning approach. Using digital tools is helpful in the pedagogical approach, as they facilitate learning. Only a third of students found digital tools better than conventional forms of learning. Students have more time to study without travelling to school and back home.	When switching to e-learning, students perceived a deterioration in the content of lectures. The transition from traditional to e-learning significantly reduced the opportunities for social interaction in the study process.
Mambwe and Tembo 2021, Zambia [32]	To explore nursing students’ e-learning experiences while pursuing a midwifery course during the pandemic.	Qualitative methodology; descriptive design—focus groups.	*n* = 60 midwifery students.	E-learning remains the quickest, broadest, most efficient and most effective way of teaching and learning, with more significant potential for being the future mainstay of education.	Students living in rural places face the challenge of poor internet connectivity due to weak signal strength. Students fail to participate during interactive virtual classes due to uncharged electronic gadgets or interrupted lectures due to power outages. Some students had neither personal computers nor a smartphone to access learning materials.
Mashaal et al. (2020), Jordan [33]	To examine the stress levels, stressors, and associated sociodemographic variables among nursing students due to the transition to e-learning during COVID-19.	Mixed-methods study; sequential explanatory design.	*n* = 355 nursing students.		Students from families with lower incomes who used smartphones to study found paying an internet subscription an additional financial burden, and students who did not have a quiet place to study experienced e-learning as more stressful.
Oducado and Soriano (2021), Philippines [29]	To examine nursing students’ attitudes towards e-learning in two selected nursing schools	Quantitative methodology; cross-sectional design.	*n* = 111 nursing students.	Nursing students had intermediate computer competency (75%) and somewhat stable internet connection (67%). Those students with long-lasting internet connections appear to have a better attitude towards e-learning.	They generally had negative (41%) and ambivalent attitudes (31%) towards e-learning. The nursing students considered e-learning impersonal and lacking feeling (80%), resulting in less student–teacher interactions (76%).
Oducado and Estoque (2021), Philippines [30]	The purpose of the study was to find out the level of stress, satisfaction, and academic performance during online learning in undergraduate nursing students.	Quantitative methodology; cross-sectional descriptive design.	*n* = 108, second-year undergraduate nursing students.		Students reported that online learning is very stressful (47%), satisfaction with online learning was low (37%), and academic performance during the COVID-19 outbreak was reported as poor (37%) to fair (50%).
Ramos-Morcillo et al. (2020), Spain [24]	To discover the learning experiences and the expectations about the educational changes in light of the abrupt transition from face-to-face to e-learning education.	Qualitative methodology; descriptive design—semi-structured interviews.	*n* = 32 nursing students.		E-learning presents limitations, especially for older students, students in rural areas, students with family obligations and work duties, and students with limited access to e-learning resources.
Terzi et al. (2021), Turkey [27]	To identify the factors affecting nursing students’ attitudes towards distance education.	Quantitative methodology; cross-sectional descriptive design.	*n* = 380 nursing students.		Distance education was not appropriate for nursing education and basic practical nursing skills. The distance education system was not operating well enough, and it was not appropriate to perform mandatory clinical practices for nursing in virtual hospitals.
Topuz et al. (2021), Turkey [28]	To determine midwifery students’ experiences with distance education.	Qualitative methodology; exploratory case study design.	*n* = 50 midwifery students.	Students were satisfied with distance education, replay capability, efficiency increase, saving time, accessibility and comfort, uninterrupted education, reduced health risk, and technological experience gain.	The limitations are the non-availability of practical courses, technological differences, inefficiency, superficial lectures, difficulties in following up the course, insufficient interaction and environmental impact.
Wallace et al. (2021), USA [31]	To understand the lived experience and perceptions of students transitioning to remote learning during the COVID-19 pandemic.	Qualitative methodology; descriptive phenomenological design.	*n* = 11 nursing students.	Students became self-directed, formed independent study groups, and practised nursing skills on items available in their learning. They appreciated the flexibility of remote learning, extra study time garnered from not having long commutes, increased time with family, and the ability to take time for self-care.	Students face technological challenges: connectivity issues, netiquette, distractions in their learning environment, uncertainty about virtual communication (when to ask questions, how to approach professors), and problems related to unfamiliarity with online teaching methods professors use. Most students described feeling isolated, lonely, missing study groups and unable to ask their peers questions. Students faced increased responsibilities outside their academic workload, resulting in role stress and strain. Influencing factors included family dynamics and structure changes, increased responsibilities such as home-schooling, and financial pressures.

**Table 2:** Critical appraisal of qualitative research.

JBI CA Checklist Question	1	2	3	4	5	6	7	8	9	10	Total Score; Quality Assessment
Including Articles (*n* = 5)											
Mambwe and Tembo 2021 [32]	Y	Y	Y	Y	N	N	N	Y	Y	Y	7/10; Medium quality
Ramos-Morsillo, et al., 2020 [24]	Y	Y	Y	Y	Y	N	N	Y	Y	Y	8/10; High quality
Topuz et al., 2021 [28]	Y	Y	Y	Y	Y	Y	Y	Y	Y	Y	10/10; High quality
Wallace et al., 2021 [31]	Y	Y	Y	Y	Y	Y	N	Y	U	Y	8/10; High quality

Note: Y = Yes, N = No, U = Uncleare. (1) Is there congruity between the stated philosophical perspective and the research methodology? (2) Is there congruity between the research methodology and the research question or objectives? (3) Is there congruity between the research methodology and the methods used to collect data? (4) Is there congruity between the research methodology and the representation and analysis of data? (5) Is there congruity between the research methodology and the interpretation of results? (6) Is there a statement locating the researcher culturally or theoretically? (7) Is the influence of the researcher on the research, and vice-versa, addressed? (8) Are participants, and their voices, adequately represented? (9) Is the research ethical according to current criteria or, for recent studies, is there evidence of ethical approval by an appropriate body? (10) Do the conclusions drawn in the research report flow from the analysis, or interpretation, of the data?

**Table 3:** Critical appraisal of analytical cross-sectional studies.

JBI CA Checklist Question	1	2	3	4	5	6	7	8	Total Score; Quality Assessment
Including Articles (*n* = 11)									
Alhassan (2020) [34]	Y	Y	Y	Y	N	N	Y	Y	6/8; High quality
El-Hamed Diab and Elgahsh (2020) [12]	Y	Y	Y	Y	N	N	Y	Y	6/8; High quality
Oducado and Soriano (2021) [29]	Y	Y	Y	Y	N	N	Y	Y	6/8; High quality
Oducado and Estoque (2021) [30]	Y	Y	N	Y	N	N	Y	Y	5/8; Medium quality
Guven Ozdemir and Sonmez (2020) [26]	Y	Y	Y	Y	N	N	Y	Y	6/8; High quality
Terzi et al. (2021) [27]	Y	Y	Y	Y	N	N	Y	Y	6/8; High quality

Note: Y = Yes, N = No. (1) Were the criteria for inclusion in the sample clearly defined? (2) Were the study subjects and the setting described in detail? (3) Was the exposure measured validly and reliably? (4) Were objective, standard criteria used for measurement of the condition? (5) Were confounding factors identified? (6) Were strategies to deal with confounding factors stated? (7) Were the outcomes measured validly and reliably? (8) Was appropriate statistical analysis used?

**Table 4:** Critical appraisal for cohort studies.

JBI CA Checklist Questions	1	2	3	4	5	6	7	8	9	10	11	Total Score; Quality Assessment
Including Articles (*n* = 1)												
García-González et al. (2021) [23]	Y	Y	Y	N	N	Y	Y	Y	Y	N	Y	8/11; Medium quality

Note: Y = Yes, N = No. (1) Were the two groups similar and recruited from the same population? (2) Were the exposures measured similarly to assign people to both exposed and unexposed groups? (3) Was the exposure measured in a valid and reliable way? (4) Were confounding factors identified? (5) Were strategies to deal with confounding factors stated? (6) Were the groups/participants free of the outcome at the start of the study (or at the moment of exposure)? (7) Were the outcomes measured in a valid and reliable way? (8) Was the follow-up time reported sufficient for outcomes to occur? (9) Was follow-up complete, and if not, were the reasons to lose to follow-up described and explored? (10) Were strategies to address incomplete follow-up utilised? (11) Was appropriate statistical analysis used?

**Table 5:** Critical appraisal of mixed-methods studies.

Study (*n* = 2)	Total Score
Mashaal et al., 2020 [33]	100%
Langegård et al., 2021 [25]	75%

**Table 6:** Synthesis of the main themes.

Main Themes	Subthemes
Advantages of e-learning	Adaptability to environmental factors
	Person-centred learning
	Safety
	Encouraging innovative study/teaching approaches
	Taking responsibility for study
	Saving time
	Positive attitude towards e-learning
Challenges/limitations of e-learning	Increased burdens
	Reduced social contacts
	Health issues
	Infrastructure/material/economic conditions
	Qualification for e-learning
	Communication challenges
Recommendations for e-learning	Technical training
	Clinical training
	Teaching methods
	Dealing with the burdens

## Data Availability

The datasets generated and/or analysed during the current study are available from the corresponding author upon reasonable request.

## References

[1] Voutilainen A., Saaranen T., Sormunen M. (2017). Conventional vs. e-learning in nursing education: A systematic review and meta-analysis. Nurse Educ. Today.

[2] Salmani N., Bagheri I., Dadgari A. (2022). Iranian nursing students experiences regarding the status of e-learning during COVID-19 pandemic. PLoS ONE.

[3] Mukhtar K., Javed K., Arooj M., Sethi A. (2020). Advantages, limitations and recommendations for online learning during COVID-19 pandemic era. Pak. J. Med. Sci..

[4] Opeyemi O.Z., Adeyemi A.A., Olajuwon T.D., Nike O., Oloruntosin B.S.O. (2019). Perception of nursing students towards online learning: A case study of lautech open and distance learning centre. Int. J. Health Sci. Res..

[5] Subedi S., Nayaju S., Subedi S., Shah S.K., Shah J.M. (2020). Impact of E-learning during COVID-19 Pandemic among Nursing Students and Teachers of Nepal. Int. J. Sci. Healthc. Res..

[6] Farooq F., Rathore F.A., Nasir S. (2020). Challenges of online medical education in Pakistan during COVID-19 pandemic. J. Coll. Physicians Surg. Pak..

[7] Newman N.A., Lattouf O.M. (2020). Coalition for medical education—A call to action: A proposition to adapt clinical medical education to meet the needs of students and other healthcare learners during COVID-19. J. Card. Surg..

[8] Bediang G., Stoll B., Geissbuhler A., Klohn A.M., Stuckelberger A., Nko’o S., Chastonay P. (2013). Computer literacy and E-learning perception in Cameroon: The case of Yaounde Faculty of Medicine and Biomedical Sciences. BMC Med. Educ..

[9] Cook D.A., Levinson A.J., Garside S., Dupras D.M., Erwin P.J., Montori V.M. (2008). Internet-Based Learning in the Health Professions: A Meta-analysis. JAMA.

[10] Iyer P., Aziz K., Ojcius D.M. (2020). Impact of COVID-19 on dental education in the United States. J. Dent. Educ..

[11] Abbasi M.S., Ahmed N., Sajjad B., Alshahrani A., Saeed S., Sarfaraz S., Alhamdan R.S., Vohra F., Abduljabbar T. (2020). E-Learning perception and satisfaction among health sciences students amid the COVID-19 pandemic. Work.

[12] El-Hamed Diab G.M.A., Elgahsh N.F. (2020). E-learning During COVID-19 Pandemic: Obstacles Faced Nursing Students and Its Effect on Their Attitudes While Applying It. Am. J. Nurs. Sci..

[13] McDonald E.W., Boulton J.L., Davis J.L. (2018). E-learning and nursing assessment skills and knowledge—An integrative review. Nurse Educ. Today.

[14] Oliveira M.M.S., Penedo A.S.T., Pereira V.S. (2018). Distance education: Advantages and disadvantages of the point of view of education and society. Dialogia.

[15] Wiederhold B.K. (2020). Connecting through Technology during the Coronavirus Disease 2019 Pandemic: Avoiding “Zoom Fatigue”. Cyberpsychol. Behav. Soc. Netw..

[16] Suleyiman M., Atinkut Z. (2018). Prevalence and associated factors of stress among undergraduate students in Ambo University: Implication for Intervention. Int. J. Psychol. Couns..

[17] Stern C., Lizarondo L., Carrier J., Godfrey C., Rieger K., Salmond S., Apostolo J., Kirkpatrick P., Loveday H. (2021). Methodological guidance for the conduct of mixed methods systematic reviews. JBI Evid. Implement..

[18] Page M.J., McKenzie J.E., Bossuyt P.M., Boutron I., Hoffmann T.C., Mulrow C.D. (2021). The PRISMA 2020 statement: An updated guideline for reporting systematic reviews. PLoS Med.

[19] Melnyk B.M., Fineout-Overholt E. (2019). Evidence-Based Practice in Nursing & Healthcare: A Guide to Best Practice.

[20] Teixeira S.M.A., Coelho J.C.F., Sequeira C.A.d.C., i Canut M.T.L., Ferré-Grau C. (2019). The effectiveness of positive mental health programs in adults: A systematic review. Health Soc. Care Community.

[21] Pluye P., Robert E., Cargo M., Bartlett G., O’cathain A., Griffiths F., Boardman F., Gagnon M.P., Rousseau M.C., Robert E. (2011). Proposal: A Mixed Methods Appraisal Tool for Systematic Mixed Studies Reviews. http://mixedmethodsappraisaltoolpublic.pbworks.com.

[22] Thomas J., Harden A. (2008). Methods for the thematic synthesis of qualitative research in systematic reviews. BMC Med. Res. Methodol..

[23] García-González J., Ruqiong W., Alarcon-Rodriguez R., Requena-Mullor M., Ding C., Ventura-Miranda M.I. (2021). Analysis of anxiety levels of nursing students because of e-learning during the COVID-19 pandemic. Healthcare.

[24] Ramos-Morcillo A.J., Leal-Costa C., Moral-García J.E., Ruzafa-Martínez M. (2020). Experiences of nursing students during the abrupt change from face-to-face to e-learning education during the first month of confinement due to COVID-19 in Spain. Int. J. Environ. Res. Public Health.

[25] Langegård U., Kiani K., Nielsen S.J., Svensson P.A. (2021). Nursing students’ experiences of a pedagogical transition from campus learning to distance learning using digital tools. BMC Nurs..

[26] Guven Ozdemir N., Sonmez M. (2020). The relationship between nursing students’ technology addiction levels and attitudes toward e-learning during the COVID-19 pandemic: A cross-sectional study. Perspect. Psychiatr. Care.

[27] Terzi B., Azizoğlu F., Özhan F. (2021). Factors affecting attitudes of nursing students towards distance education during the COVID-19 pandemic: A web-based cross-sectional survey. Perspect. Psychiatr. Care.

[28] Topuz Ş., Yilmaz Sezer N., Aker M.N., Gönenç İ.M., Öner Cengiz H., Er Korucu A. (2021). A SWOT analysis of the opinions of midwifery students about distance education during the COVID-19 pandemic a qualitative study. Midwifery.

[29] Oducado R.M.F., Soriano G.P. (2021). Shifting the education paradigm amid the COVID-19 pandemic: Nursing students’ attitude to e-learning. Afr. J. Nurs. Midwifery.

[30] Oducado R.M.F., Estoque H. (2021). Online Learning in Nursing Education During the COVID-19 Pandemic: Stress, Satisfaction, and Academic Performance. J. Nurs. Pract..

[31] Wallace S., Schuler M.S., Kaulback M., Hunt K., Baker M. (2021). Nursing student experiences of remote learning during the COVID-19 pandemic. Nurs. Forum.

[32] Mambwe P., Tembo J. (2021). Exploring Students’ Experiences of E-learning in Midwifery Course: A Qualitative Study Involving Nursing Students Taking Midwifery Course at Rusungu University. Texila Int. J. Nurs..

[33] Mashaal D., Rababa M., Shahrour G. (2020). Distance learning-related stress among undergraduate nursing students during the COVID-19 pandemic. J. Nurs. Educ..

[34] Alhassan R.K. (2020). Assessing the preparedness and feasibility of an e-learning pilot project for university level health trainees in Ghana: A cross-sectional descriptive survey. BMC Med. Educ..

[35] Dewart G., Corcoran L., Lorraine T., Petrovic K. (2020). Nursing education in a pandemic: Academic challenges in response to COVID-19. Nurse Educ. Today.

[36] Monforte-Royo C., Fuster P. (2020). Coronials: Nurses who graduated during the COVID-19 pandemic. Will they be better nurses?. Nurse Educ. Today.

[37] Posey L., Pintz C. (2017). Transitioning a bachelor of science in nursing program to blended learning: Successes, challenges & outcomes. Nurse Educ. Pract..

[38] Alsabawy A.Y., Cater-Steel A., Soar J. (2016). Determinants of perceived usefulness of e-learning systems. Comput. Human. Behav..

[39] Ruiz-Grao M.C., Cebada-Sánchez S., Ortega-Martínez C., Alfaro-Espín A., Candel-Parra E., García-Alcaraz F., Molina-Alarcón M., Delicado-Useros V. (2022). Nursing Student Satisfaction with the Teaching Methodology Followed during the COVID-19 Pandemic. Healthcare.

[40] Prosen M., Karnjuš I., Ličen S. (2022). Evaluation of E-Learning Experience among Health and Allied Health Professions Students during the COVID-19 Pandemic in Slovenia: An Instrument Development and Validation Study. Int. J. Environ. Res. Public Health.

[41] Bahrambeygi F., Shojaeizadeh D., Sadeghi R., Nasiri S., Ghazanchaei E. (2018). The Effectiveness of an E-Learning Program on Nurse’s Knowledge and Behavior for Caring of Patients with Thromboembolism: A Comparative Study. J. Nurs. Healthc. Manag..

[42] Kattoua T., Al-Lozi M., Alrowwad A. (2013). A Review of Literature on Knowledge Management using ICT in Higher Education. Int. J. Bus. Manag. Econ. Res..

[43] Moore R.L. (2014). Importance of Developing Community in Distance Education Courses. TechTrends.

[44] Bdair I.A. (2021). Nursing students’ and faculty members’ perspectives about online learning during COVID-19 pandemic: A qualitative study. Teach. Learn. Nurs..

[45] Singh H.K., Joshi A., Malepati R.N., Najeeb S., Balakrishna P., Pannerselvam N.K., Singh Y.K., Ganne P. (2021). A survey of E-learning methods in nursing and medical education during COVID-19 pandemic in India. Nurse Educ. Today.

[46] Swaminathan N., Govindharaj P., Jagadeesh N.S., Ravichandran L. (2021). Evaluating the effectiveness of an online faculty development programme for nurse educators about remote teaching during COVID-19. J. Taibah Univ. Med. Sci..

[47] Tomić S.D., Tomić S., Malenković G., Malenković J., Šljivo A., Mujičić E. (2023). COVID-19-Related Stress, Fear and Online Teaching Satisfaction among Nursing Students during the COVID-19 Pandemic. Healthcare.

[48] Ortega-Donaire L., Bailén-Expósito J., Álvarez-García C., López-Medina I.M., Álvarez-Nieto C., Sanz-Martos S. (2023). Satisfaction of Online University Education during the COVID-19 Pandemic. Healthcare.

[49] Savitsky B., Findling Y., Ereli A., Hendel T. (2020). Anxiety and coping strategies among nursing students during the COVID-19 pandemic. Nurse Educ. Pract..

